# Correction: Knowledge of Thai women in cervical cancer etiology and screening

**DOI:** 10.1371/journal.pone.0318184

**Published:** 2025-01-23

**Authors:** Uraiwan Khomphaiboonkij, Nattapong Sreamsukcharoenchai, Supakorn Pitakkarnkul, Kristsanamon Rittiluechai, Siriwan Tangjitgamol, Thai Gynecologic Cancer Research Group

Thai Gynecologic Cancer Research Group should be included in the author byline. Thai Gynecologic Cancer Research Group should be listed as the sixth author and affiliated with #5:Thai Gynecologic Cancer Society, Bangkok, Thailand. The contributions of this author are as follows: formulated research goals, maintained research data, applied formal techniques to analyze data, secured financial support, conducted investigation process, developed the methodology, managed the research activity planning and execution, provided study materials, led the planning and execution of research activities, verified the results, prepared data presentation, created and presented the initial draft, critical review and revision.

The correct citation is: Khomphaiboonkij U, Sreamsukcharoenchai N, Pitakkarnkul S, Rittiluechai K, Tangjitgamol S, Thai Gynecologic Cancer Research Group (2023) Knowledge of Thai women in cervical cancer etiology and screening. PLoS ONE 18(5): e0286011. https://doi.org/10.1371/journal.pone.0286011

The following information is missing from the Funding statement: This study is funded by the Thai Gynecologic Cancer Society Research Fund.

There are errors in Table 3. The negative signs (-) in the "Correct," "Wrong," and "Unsure" columns should have not been indicated. Please see the correct Table 3 here.

**Table 3:** Questions and answers about knowledge of cervical cancer screening and HPV (N = 499)

Knowledge	Correct		Wrong		Unsure	
	n	(%)	n	(%)	n	(%)
**Knowledge about cervical cancer screening (N = 499)**						
1. All women should be screening.	20	4	476	95.4	3	0.6
2. Screening should begin at age 25.	345	69.1	144	28.9	10	2
3. Women who have never had sexual intercourse may begin the screening at age 30-year-old.	273	54.7	204	40.9	22	4.4
4. If the results are normal, re-screening is done every 2–3 years or 5 years, depending on the screening method.	351	70.3	132	26.5	16	3.2
5. Women who have their uterus removed along with the ovaries due to ovarian cancer still need continual screening.	87	17.4	386	77.4	26	5.2
6. Women over 65 do not need further screening after consecutive normal screening results.	181	36.3	287	57.5	31	6.2
7. There are several methods used for screening.	394	79	84	16.8	21	4.2
Knowledge	Correct		Wrong		Unsure	
	n	(%)	n	(%)	n	(%)
**Knowledge about cervical cancer screening (N = 499)**						
8. Cervical cancer screening is recommended along with an annual pelvic examination	36	7.2	461	92.4	2	0.4
9. Screening can be done by naked eye examination by a doctor/health worker after applying vinegar on the cervix.	158	31.7	301	60.3	40	8
10. Screening can be done by cervical cytologic examination.	468	93.8	24	4.8	7	1.4
11. Screening can be done by HPV testing.	445	89.2	41	8.2	13	2.6
12. Screening can be done by cervical cytologic examination and HPV testing	461	92.4	25	5	13	2.6
13. Screening can be done by ultrasound and computer tomography.	185	37.1	288	57.7	26	5.2
14. Screening can be done by testing the blood for cancer markers.	148	29.7	326	65.3	25	5
Knowledge about HPV (N = 389*)						
15. HPV infection is preventable	360	92.5	24	6.2	5	1.3
16. HPV infection can be prevented by avoiding sexual activity	193	49.6	180	46.3	16	4.1
17. HPV infection can be prevented by vaccination	331	85.1	49	12.6	9	2.3
18. Venereal wart is due to non-oncogenic types of HPV	196	50.4	165	42.4	28	7.2
19. Cervical cancer is due to oncogenic types of HPV	352	90.5	23	5.9	14	3.6
*Excluding 110 participants who had never heard about HPV						
