# Supplementary material for: Knowledge of Thai women in cervical cancer etiology and screening
Source: PLoS One. 2023 May 18;18(5):e0286011. doi: 10.1371/journal.pone.0286011 (PMC10194861; doi:10.1371/journal.pone.0286011)
Supplement: S1 Table — (DOCX) [file pone.0286011.s001.docx]

**S1 Table.** Content validity index of questionnaire about knowledge of cervical cancer screening and HPV
